# GASTRIC NEUROENDOCRINE TUMOR: REVIEW AND UPDATE

**DOI:** 10.1590/0102-6720201700020016

**Published:** 2017

**Authors:** Andre Roncon DIAS, Beatriz Camargo AZEVEDO, Luciana Bastos Valente ALBAN, Osmar Kenji YAGI, Marcus Fernando Kodama Pertille RAMOS, Carlos Eduardo JACOB, Leandro Cardoso BARCHI, Ivan CECCONELLO, Ulysses RIBEIRO-JR, Bruno ZILBERSTEIN

**Affiliations:** 1 Cancer Institute of São Paulo State; 2 School of Medicine, University of São Paulo, São Paulo, SP, Brazil.

**Keywords:** Gastric carcinoid, Gastric neuroendocrine tumor, Treatment, review

## Abstract

**Introduction:**

The frequency of gastric neuroendocrine tumors is increasing. Reasons are the popularization of endoscopy and its technical refinements. Despite this, they are still poorly understood and have complex management.

**Aim:**

Update the knowledge on gastric neuroendocrine tumor and expose the future perspectives on the diagnosis and treatment of this disease.

**Method:**

Literature review using the following databases: Medline/PubMed, Cochrane Library and SciELO. Search terms were: gastric carcinoid, gastric neuroendocrine tumor, treatment. From the selected articles, 38 were included in this review.

**Results:**

Gastric neuroendocrine tumors are classified in four clinical types. Correct identification of the clinical type and histological grade is fundamental, since treatment varies accordingly and defines survival.

**Conclusion:**

Gastric neuroendocrine tumors comprise different subtypes with distinct management and prognosis. Correct identification allows for a tailored therapy. Further studies will clarify the diseases biology and improve its treatment.

## RESUMO

**Introdução:**

A frequência de tumores neuroendócrinos gástricos está aumentando. As razões são a popularização da endoscopia e seus refinamentos técnicos. Apesar disso, os gástricos ainda são pouco compreendidos e têm manejo complexo.

**Objetivo:**

Atualizar os conhecimentos nos tumores neuroendócrinos gástricos e expor as perspectivas futuras no diagnóstico e tratamento.

**Método:**

Revisão da literatura utilizando as seguintes bases de dados: Medline/PubMed, Cochrane Library e SciELO. Os descritores da busca foram: carcinóide gástrico, tumor neuroendócrino gástrico, tratamento. Dos artigos selecionados, 38 foram incluídos nesta revisão.

**Resultados:**

Tumores neuroendócrinos gástricos são classificados em quatro tipos clínicos. A identificação correta do tipo clínico e grau histológico é fundamental, pois a conduta é variável e define a sobrevida.

**Conclusão:**

Tumor neuroendócrino gástrico possui diferentes subtipos com tratamento e prognóstico distintos. A identificação correta destes e seu entendimento permite o tratamento individualizado. Estudos futuros ajudarão a esclarecer a biologia desta doença e melhorar o tratamento.

## INTRODUCTION

Gastric neuroendocrine tumors (gNETs) are neoplasms derived from the enterochromaffin-like cells (ECL cells) of the gastric mucosa. They are rare lesions with an indolent behavior and neuroendocrine differentiation. Although uncommon, their diagnosis is increasing, due to the widespread use of upper digestive endoscopy and the technical refinement of endoscopists^4^.

The ECL cells are avid for silver salts and play a fundamental role in the regulation of acid secretion. Following food intake, the G cells of the antrum secrete gastrin that stimulates the ECL cells and the histamine-producing parietal cells to secrete hydrochloric acid (HCL). Negative feed-back comes from the D cells, which are stimulated by the HCL and secrete somatostatin who acts reducing the secretion of gastrin^4^.

**FIGURE 1:**
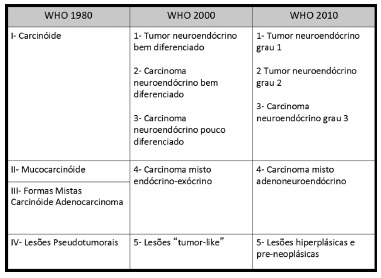
Evolution in gNETs terminology

It is essential to understand these mechanisms to classify gastric gNETs in four clinical subtypes, with distinct treatment management and prognosis^4^ ^,^ ^12^ ^,^ ^27^. It is also important to emphasize that this classification is different from the three histological grades proposed by the World Health Organization (WHO). Furthermore, the WHO terminology for gNETs underwent changes in recent years, which amplified the difficulty to understand this complex disease (Figure 1).

## METHOD

Literature review using the following databases: Medline/PubMed, Cochrane Library and SciELO. Search terms were: gastric carcinoid, gastric neuroendocrine tumor, treatment. Articles in English and Portuguese were considered.

## RESULTS

Classification and diagnosis

### Type I

Type I lesions correspond to the majority of gNETs found in the stomach (70-80%) and they are associated with autoimmune chronic atrophic gastritis. The patient has anti-parietal cell or anti-intrinsic factor antibodies, leading to the destruction of the gastric parietal cell, reducing the level of HCL (achlorhydria), consequently increasing the gastrin production by G cells (hypergastrinemia)^12^ ^,^ ^21^. This hormone excess promotes ECL cells hyperplasia, favoring the appearance of multiple small lesions, usually with little aggressive behavior and good prognosis^21^.

A decrease in the intrinsic factor with reduction on vitamin B12 absorption also occurs leading to macrocytic anemia (pernicious or megaloblastic)^15^.

**FIGURE 2:**
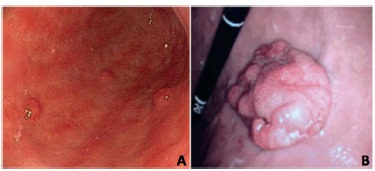
Endoscopic images: A) showing chronic atrophic pangastritis with three low grade gNETs; B) with solitary gNET type III

Diagnosis of type I is made by upper gastrointestinal (GI) endoscopy with biopsy. Endoscopic findings consist of pale, yellowish and transparent blood vessels of the antral mucosa, contrasting with the smooth and reddish mucosa of normal areas. Neuroendocrine tumors are visualized as small, reddish polyps and often being multiple (Figure 2A).

Histological examination shows atrophy of the mucosa cells, absence of parietal cells and neuroendocrine cell hyperplasia. It also confirms the diagnosis of NET. Increased serum gastrin and low serum vitamin B12 are usually observed. Anti-parietal cell and anti-intrinsic factor antibodies may also be present^12^ ^,^ ^15^ ^,^ ^18^. Finally, the gastric acidity dosage reveals high pH (pH≥7)^22^ ^,^ ^32^.

### Type II

Type II lesions are caused by gastrinomas (gastrin-producing tumors), also known as Zollinger-Ellison syndrome. In most cases, the patient has multiple endocrine neoplasia type I (MEN-1) and should be investigated with serum sequencing for MEN1 gene^16^.

The frequency of type II gNETs is around 7% and the lesions are usually small and multiple. The metastatic potential is also low, although higher than in type I^4^ ^,^ ^12^.

For diagnostic confirmation, upper GI endoscopy with gastric biopsy reveals normal or hypertrophic gastric mucosa^29^. Hypergastrinemia and gastric pH<2 (hyperchlorhydria) are observed. Serial measurement of gastrin levels following intravenous administration of secretin can also be performed revealing an increase in gastrin levels for patients with gastrinoma, whereas they decrease in healthy individuals^3^.

After confirming the diagnosis, research should continue aiming to localize the gastrinoma and, if possible, remove it surgically. Most of these lesions are located in the triangle of gastrinomas determined by the junction of the cystic duct with the common hepatic duct, the transition from second to third duodenal portions and the pancreatic cervix^33^. Computed tomography (CT) scan, magnetic resonance imaging (MRI), endoscopic ultrasound, scintigraphy with octreotide, selective angiography, positron emission tomography and intraoperative ultrasonography are usefull methods that help finding the lesion.

### Type III

Type III gNETs consist of a sporadic lesion and has the greatest potential to generate metastasis. The survival of these patients is also worse (75-80% at five years compared to 90-95% for type I)^4^ ^,^ ^12^. Generally, the lesion is unique and greater than 1 cm, with normal gastrinemia.

Diagnosis is made by upper GI endoscopy with biopsy, observing a single lesion in normal gastric mucosa (Figure 2B). Although rare, carcinoid syndrome (due to the presence of liver metastasis) can be the initial manifestation.

### Type IV

It is worth mentioning that three recent reports suggest a fourth type of gNET. It consists of multiple small lesions and the histological examination reveals hypertrophy and hyperplasia of parietal cells with vacuolated cytoplasm. A structural abnormality prevents the HCL, produced by these cells, from being secreted. Consequently, achlorhydria, hypergastrinemia and hyperplasia of neuroendocrine cells occur^1^ ^,^ ^23^ ^,^ ^27^.

## Immunohistochemistry

**FIGURE 3:**
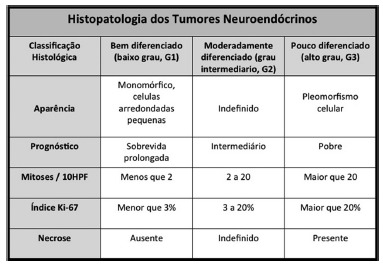
Classification according to histological grades

**FIGURE 4:**
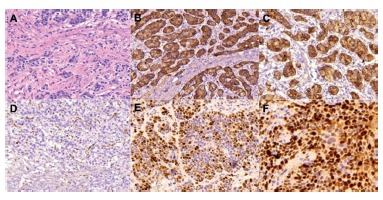
Histological sections of well-differentiated gNET (x 200): A) hematoxylin-eosin; B) positivity for chromogranin A; C) positivity for synaptophysin; D) Ki-67 nuclear proliferative index <2%; E) Ki-67 of 50-60%; F) Ki-67 of 70-80%.

Immunohistochemical analysis is essential in NETs. It allows diagnostic confirmation and permits classifying the lesion according to the histological grades defined by the WHO (Figure 3)^8^ ^,^ ^30^. For diagnostic confirmation chromogranin A and synaptophysin are necessary, while for prognostic definition the proliferative index Ki-67 and the number of mitoses per high magnification field are required (Figure 4)^8^ ^,^ ^30^. Other markers, such as p53, have being studied, and also relates to prognosis and risk of metastasis^31^.

## Staging

CT scan of the abdomen is recommended for type I and II gNETs larger than 2 cm and for all type III lesions. MRI of the abdomen, octreotide scintigraphy and PET-CT may be usefull in specific cases^34^. 

### Treatment

**FIGURE 5:**
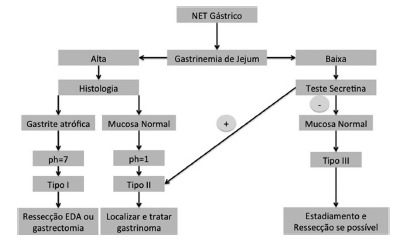
Treatment algorithm

The treatment of gNETs depends on the clinical type, disease extent, the differentiation of the lesion and the presence or absence of poor prognostic factors (Figure 5). Accordingly to the WHO, these neoplasms are classified into three histologic degrees with distinct prognosis (Figure 3)^30^.

Poor prognosis factors are: lesion ≥2 cm; deep submucosa invasion or beyond (at least 24% are metastatic); Ki-67 ≥3%; vascular invasion; low degree of structural differentiation; presence of atypia and/or necrosis.

Carcinoid crisis should also be prevented before and after any tumor manipulation or anesthesia. This may be accomplished by the administration of intravenous or subcutaneous octreotide^35^ ^9^.

### Type I

As most of these lesions are small, well-differentiated and with excellent prognosis, treatment usually consists in the serial endoscopic resection of these lesions^12^. Supplementation of vitamin B12 is also recommended.

Surgical treatment is necessary only when endoscopic resection is not feasible or when poor prognostic factors are present. While the surgical indication is obvious when deep invasion is observed, for those cases with lymph node metastasis or for lesions not suitable for endoscopic resection, there is no evidence that supports or not surgery when there is only necrosis, vascular invasion or an elevated Ki-67. A recent article validated the WHO classification, demonstrating the presence of a lymph node metastasis in a patient with a small and superficial type I gNET whose only poor prognostic factor was a Ki-67 of 7% (G2 according to the WHO grading)^19^. This also highlights the need for diligent analysis of all resected lymph node. Our group recommends Carnoy`s solution as specimen fixative to improve lymph node detection^9^ ^,^ ^28^. 

It is also unclear when surgery should be indicated for patients with frequent recurrences or when there is a high number of lesions. At this time there is no evidence in the literature that allows a strong recommendation, since there is no consensus of what is a “frequent recurrence” or a “high number” of lesions. Thus, management of these cases should be tailored and discussed with the patient.

The best operation for type I gNETs is also controversial^37^. Antrectomy has been proposed to remove the gastrin-producing G cells; however, failure may occur due to improper removal of these cells or because the ECL cells became autonomous. For these reasons subtotal or total gastrectomy are more suitable options. Subtotal gastrectomy allows adequate removal of G cells, while total gastrectomy is reserved for those cases with substantial disease in the gastric fundus^3^ ^,^ ^7^ ^,^ ^12^. Concerning lymphadenectomy, it should be performed when there is any evidence of extra gastric disease or when poor prognosis factors are present. Its extension is not stablished in the literature, option for D1, D1+ or D2 should be performed on an individual basis. Minimally invasive procedures are adequate for these patients^2^.

It is worth mentioning the clinical treatment of type I gNETs, although this will hardly be an effective option in the long term. Some authors used somatostatin analogues (octreotide) to decrease gastrinemia in small groups of patients. However, after discontinuation of the treatment serum gastrin rose again in a one year follow-up, although no new lesions were observed in the short term^13^ ^,^ ^17^. Therefore, this treatment should be reserved for those patients unfit for surgical resection.

### Type II

Treatment of type II gNETs consists in localizing and resecting the gastrinoma. As for gastric lesions, unless there is some factor of poor prognosis, endoscopic resection is enough. 

### Type III

These lesions should be managed aggressively with total or subtotal gastrectomy (depending on location) associated with lymphadenectomy.

If there is resectable metastatic disease, it should also be treated. For unresectable liver disease, local therapies such as arterial embolization or radioablation have a success rate of 50%^14^ ^,^ ^20^. If there is extrahepatic metastasis or recurrent symptomatic disease, systemic therapy with cytotoxic chemotherapy (streptozocin combined with 5-fluorouracil or cyclophosphamide, doxorubicin mono drug or with 5-fluorouracil, dacarbazine or temozolamide, oxaliplatin with capecitabine or 5-flurouracil with leucovorin) or molecular targeted agents (bevacizumab, sorafenib, sunitinib, pazopanib and everolimus) can be introduced. The goal is to keep the disease stable, with a small gain in progression-free survival, since the response rate is very low and there is no evidence of gain in overall survival. Preferably, these patients should be included in randomized clinical studies^24^.

## Carcinoid syndrome

This is a rare event in gNETs and its clinical manifestation is atypical consisting exclusively of redness due to histamine production^26^. Symptoms control is achieved with somatostatin analogs (octreotide or lanreotide) and interferon alfa in low doses for refractory cases^10^ ^,^ ^26^.

## Follow up

Disease evolution is quite heterogeneous, with a median survival ranging from 13 months to more than 10 years^36^.

The recommendation of the National Comprehensive Cancer Network (NCCN) for follow-up consists of anamnesis, physical examination, upper GI endoscopy, abdominal CT scan or MRI and serum chromogranin A, every six months for 1-2 years, annually for four more years and then every two years until 10 years after surgery^24^. Dosage of urinary 5-hydroxyindoleacetic acid is not recommended since gNETs do not produce serotonin^26^. Types I and II lesions <2 cm and without poor prognostic factors may be followed only with anamnesis, physical examination and upper GI endoscopy every 6-12 months^24^.

Serum chromogranin A is used as a prognostic factor and response marker for chemotherapy^3^. However, it should be analyzed with caution due to its low specificity, for instance: somatostatin analogs and proton pump inhibitors may alter it^6^.

## Trends

The presence of somatostatin receptors in NETs have been used as starting point for the development of new diagnostic tests and therapeutic methods. A cheaper and faster octreotide scintigraphy has been obtained after labeling it with technetium instead of indium^25^. Another recent innovation is the use of gallium marked octreotide, which can be captured by positron emission tomography scans (PET and PET-CT). Recent data showed undoubted superiority of this method over the octreotide scintigraphy and probably it will become the new standard in detecting NETs^11^ ^,^ ^26^.

Somatostatin analogues have also been utilized as carriers of radioactive molecules. This allows specific treatment of NET cells expressing somatostatin receptors, minimizing side effects and maximizing results. Currently, this therapy is only available in few specialized centers^5^. 

New serum markers that help the diagnosis are also being studied. Serum enolase which is most often released by aggressive undifferentiated tumors is an example. From this knowledge more specific follow-up tests for each histological tumor type may be achieved^24^.

## CONCLUSIONS

Gastric NETs consist of a complex disease that includes different subtypes with distinct management and prognosis. Correct identification of the clinical type and histological grade allows for a tailored management. Further studies will clarify the diseases biology and improve its treatment.
